# A Randomized, Non-Inferiority Study Comparing Efficacy and Safety of a Single Dose of Pegfilgrastim versus Daily Filgrastim in Pediatric Patients after Autologous Peripheral Blood Stem Cell Transplant

**DOI:** 10.1371/journal.pone.0053252

**Published:** 2013-01-07

**Authors:** Simone Cesaro, Francesca Nesi, Gloria Tridello, Massimo Abate, Irene Sara Panizzolo, Rita Balter, Elisabetta Calore

**Affiliations:** 1 Pediatric Hematology Oncology, Azienda Ospedaliera Universitaria Integrata, Verona, Italy; 2 Pediatric Hematology Oncology, Ospedale Infantile Regina Margherita, Torino, Italy; 3 Chemotherapy Unit, Istituto Ortopedico Rizzoli, Bologna, Italy; 4 Department of Pediatrics, Pediatric Hematology Oncology, Padova, Italy; Robert Wood Johnson Medical School, United States of America

## Abstract

**Purpose:**

To assess the non-inferiority of pegfilgrastim versus filgrastim in speeding the recovery of polymorphonuclear cells (PMN) in pediatric patients who underwent autologous peripheral blood stem cell transplant (PBSCT).

**Methods:**

The sample size of this randomized, multicenter, phase III study, was calculated assuming that a single dose of pegfilgrastim of 100 ug/kg was not inferior to 9 doses of filgrastim of 5 ug/kg/day. Randomization was performed by a computer-generated list and stored by sequentially numbered sealed envelopes.

**Results:**

Sixty-one patients, with a median age of 11.5 years, were recruited: 29 in the filgrastim arm and 32 in the pegfilgrastim arm. Twenty percent were affected by lymphoma/leukaemia and eighty percent by solid tumors. The mean time to PMN engraftment was 10.48 days (standard deviation [SD] 1.57) and 10.44 days (SD 2.44) in the filgrastim and pegfilgrastim arms, respectively. Having fixed a non-inferiority margin Delta of 3, the primary endpoint of non-inferiority was reached. No differences were observed for other secondary endpoints: platelet engraftment, mean time to platelet recovery (28 days vs. 33 days), fever of unknown origin (79% vs. 78%), proven infection (34% vs. 28%), mucositis (76% vs. 59%). After a median follow-up of 2.3 years (95% C.I.: 1.5, 3.3), 20 deaths were observed due to disease progression.

**Conclusions:**

We conclude that pegfilgrastim was not inferior to daily filgrastim in pediatric patients who underwent PBSCT.

**EU Clinical Trial Register Number:**

2007-001430-14

## Introduction

In autologous transplantation in the last 2 decades, peripheral blood stem cells (PBSC) have progressively become the preferred source of stem cells in place of bone marrow cells [1]. The most important reason is their capability to shorten the period of aplasia, accelerating neutrophil recovery and reducing infectious morbidity. Notwithstanding that myeloid engraftment may be influenced by the quality and quantity of progenitor cells, the use of granulocyte-colony stimulating factor (G-CSF) is recommended for autologous PBSC, regardless of the number of CD34+/kg of patient body weight infused [2]. Most retrospective and prospective studies have confirmed that the use of G-CSF reduced the period of severe neutropenia compared to untreated controls or placebo, without affecting platelet engraftment; moreover, most of randomized prospective studies found additional advantages in reduction of days of intravenous administration of antibiotics and length of hospitalization [3],[4]. The choice of G-CSF, filgrastim, lenograstim, and more recently biosimilars is left to the physician's discretion because they are considered equally efficacious; but the availability of pegfilgrastim, the pegylated form of filgrastim that has a longer half-life, make it possible to cover the entire period of aplasia with just a single injection. As shown in a recent meta-analysis, the use of pegfilgrastim is attractive because it has been associated with clinical advantages in terms of a shorter duration of severe neutropenia and of febrile neutropenic episodes [5].

All these studies were performed in adult patients whereas there are limited data regarding the use of pegfilgrastim in pediatric patients. We report the results of a prospective, randomized study assessing the non-inferiority of pegfilgrastim versus filgrastim as support agent for pediatric PBSC transplant.

## Materials and Methods

The protocol for this trial and supporting CONSORT checklist are available as supporting information; see Checklist S1 and Protocol S1.

### Patients

This was a prospective, randomized, open label, phase III, non-inferiority study, designed by the working group for supportive care of the Italian Association of Pediatric Hematology Oncology (AIEOP) that was conducted in four transplant centres from May 2007 to June 2011. The main endpoint was the hypothesis that a single dose of pegfilgrastim of 100 ug/kg (maximun 6 mg) was not inferior to 9 or more doses of filgrastim of 5 ug/kg/day (maximum 300 ug/day) in speeding recovery of PMN. Both drugs were administered beginning from day +3 after PBSC infusion. The doses of pegfilgrastim and filgrastim, and timing of their administration, were chosen on the basis of previous pediatric studies regarding the off-label use of pegfilgrastim for stem cell mobilization or prophylaxis of severe neutropenia after chemotherapy and the use of filgrastim after autologous stem cell transplantation [6]–[9],[10]–[15]. The secondary endpoints were the time to platelet engraftment, the incidence and severity of mucositis according to World Health Organization (WHO) score, the incidence of febrile neutropenia and proven infection, the duration of parenteral nutrition and intravenous antibiotic therapy, the duration of hospitalization, and overall survival. Eligible patients were aged between 0–17 years, affected by leukemia, lymphoma or solid tumor who underwent a first autologous PBSC transplant.

The study was registered at European Clinical Trial Register (Eudract number 2007-001430-14), approved by each Ethics Committee of participating centres, and all parents or patients (where applicable) gave their written informed consent before entering the study. Follow-up data are as at December 2011.

### Transplant procedures and definitions

Recruited patients were randomly assigned to the treatment arm, pegfilgrastim versus filgrastim, in the period between admission for transplant and the day of PBSC infusion (day 0). Myeloablation followed by autologous PBSC infusion was performed in high-efficiency particulate- air rooms or isolation rooms according the policy of each centre. Standard supportive care and prophylactic measures were adopted to prevent infectious complications during the neutropenic phase, i.e., fluconazole for anti-fungal prophylaxis, acyclovir and cotrimoxazole for prophylaxis of HSV and *Pneumocystis* infections, respectively. Fever, defined as the presence of an oral or axillary temperature >38.5°C in a single measurement, or >38.0°C on two or more occasions taken at least 1 hour apart, was treated empirically with broad spectrum antibiotics.

Erythrocyte and platelet products were filtered to remove leukocytes and irradiated (25 Gray). PMN and PLT recovery were defined as the first of 3 and 7 consecutive days in which the counts were >0.5×10^3^/l and 50×10^9^/l (and unsupported by transfusion), respectively.

### Statistical analysis

To calculate the sample size we assumed that the mean time of PMN engraftment was 11 days for patients treated with filgrastim (control arm), as reported in a previous AIEOP randomized study [7]. Considering a standard deviation of 3.5, we hypothesised that the time to PMN engraftment in patients treated with pegfilgrastim (experimental group) was not longer than the non-inferior margin (Delta) of 3 days compared to the control group. Considering a beta = 0.1 and an alpha = 0.05, a total of 60 patients were needed to verify this hypothesis.

To verify the primary endpoint, the 95% confidence interval (CI) of the difference between the two arms (experimental and standard) was considered. Being the delta (experimental – standard) set at 3 days, the non-inferiority is established if the upper limit of the difference in means of the 95% CI is smaller than delta, as a shorter time to PMN is considered as a better outcome. The confidence interval will be computed according to the student's t distribution.

A computer-generated randomisation list was drawn up at Data Office Centre of AIEOP in Bologna, Italy, by a statistician not involved in patient management. Simple randomization was used. The list was stored by sequentially numbered sealed envelopes that was concealed to investigators until the completion of recruitment. The local investigator, after written informed consent of parents, assigned each eligible patient to randomization list by phoning to AIEOP Data Office Centre.

Information was collected by a specific case report form containing information on demographics (sex, age), disease (type, date of diagnosis, remission status), type of mobilizing chemotherapy and complications (occurrence and duration of severe neutropenia, mucositis and infections) and PBSC transplant (type of conditioning regimen, CD34+ cells infused, PMN and PLT engraftment, early post-transplant complications, mucositis, infection, follow-up); for patients who died, date and cause of death were also recorded. To calculate early (≤100 days) post-transplant overall survival and transplant-related mortality, death by any cause and death by toxic complications were used. Descriptive statistics were reported as percentages for categorical variables and median and ranges for continuous variables. Characteristics of patients whose mobilization was successful were compared with patients whose mobilization failed using chi-square or Fisher's exact test (as appropriate) in the case of discrete variables or the Mann-Whitney test, in the case of continuous variables. The level of significance was set at 0.05. The 1-year overall survival was computed using the Kaplan Meier estimator. Median follow-up was calculated according to the inverted Kaplan-Meier technique [16].

## Results

**Figure 1:**
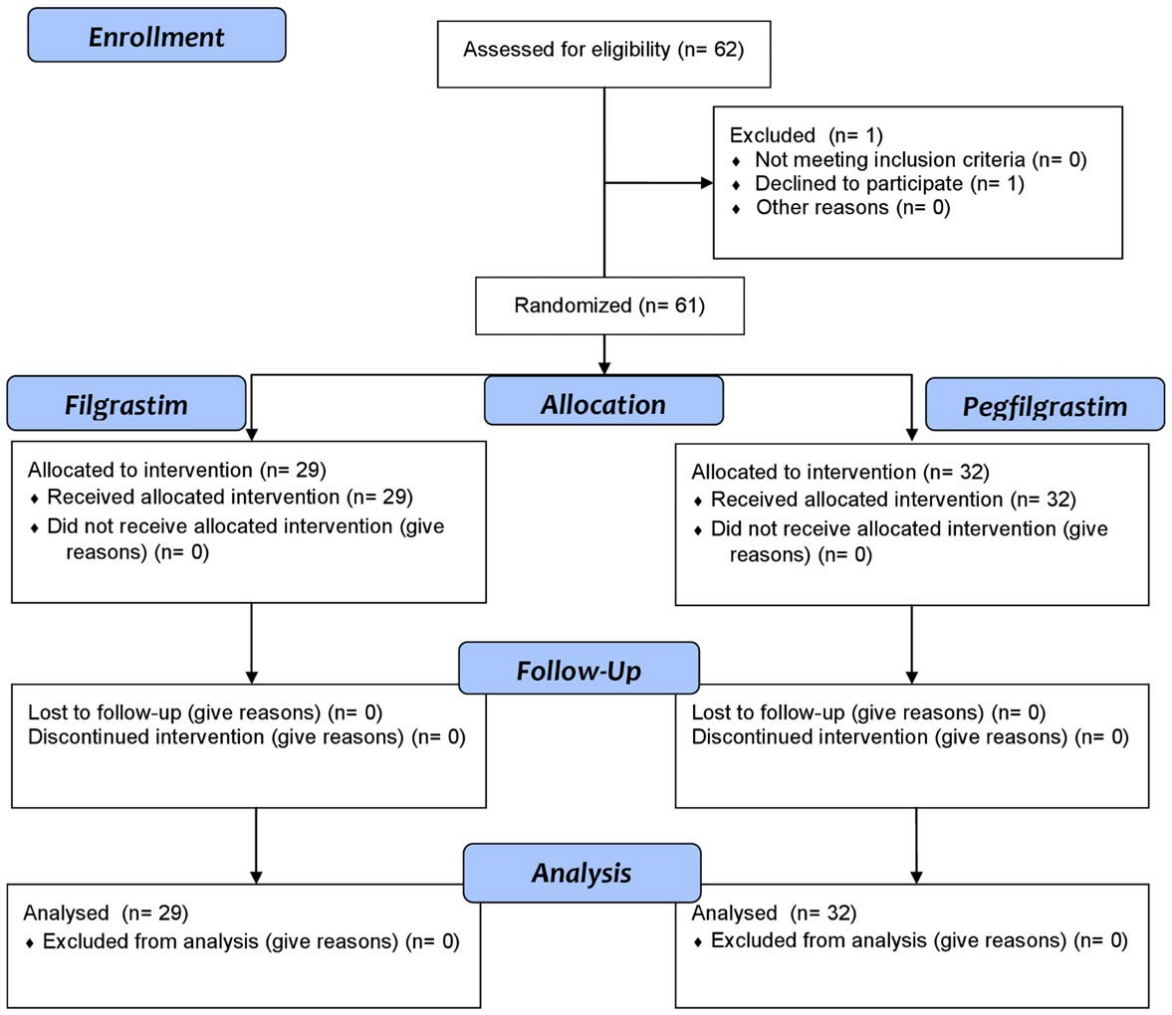
CONSORT 2010 Flow Diagram.

During the study period 61 eligible patients were enrolled, 38 (62%) males and 23 (38%) females with a median age at diagnosis of 10.5, range 1.1–16.8. Figure 1 shows the progress of the patients through the phases of the study. Twenty-nine patients were assigned by randomization to filgrastim (control arm) whilst 32 patients were assigned to pegfilgrastim (experimental arm). Twenty percent of the patients were affected by leukemia and lymphoma: acute lymphoblastic leukaemia (ALL), 3, non-Hodgkin lymphoma (NHL), 4, and Hodgkin lymphoma (HD), 5, whilst the remaining patients were affected by a solid tumor: neuroblastoma 10, Ewing sarcoma/Peripheral neutroectodermal tumor, 27, medulloblastoma, 5, Wilms tumor, 3; central nervous system tumor, 4. The main demographic and patient clinical characteristics before PBSC transplant are shown in Table 1. No differences were found due to gender, diagnosis, status of the disease at transplant, age at transplant, and body weight.

**Table 1:** The main demographic and clinical characteristics of two groups are shown according to treatment arm.

	Filgrastim N = 29 (%)	Peg-filgrastim N = 32 (%)	p
**Gender**			
**Male**	17 (58.6)	21 (65.6)	0.6
**Female**	12 (41.4)	11 (34.4)	
**Body weight**			
**Median**	38.0	31.8	0.8
**Range**	9.6–103.0	11.0–106.0	
**Underlying disease**			
**Leukemia/Lymphoma**	5 (17.2)	7 (21.9)	0.6
**Solid tumors**	24 (82.8)	25 (78.1)	
**Status of underlying disease at transplant**			
**Complete remission**	18 (62.1)	14 (43.8)	0.2
* **Other status**	11 (37.9)	18 (56.3)	
**Age at diagnosis (years)**			
**Median**	11.1	9.2	0.8
**Range**	1.1–16.8	1.4–16.8	
**Age at SCT (years)**			
**Median**	11.9	11.1	0.9
**Range**	1.6–17.2	1.7–17.4	
**Time from diagnosis to transplant (days)**			
**Median**	216.0	249.5	0.4
**Range**	67.0–1520.0	102.0–1136.0	

* other status comprised very good partial remission (16), partial remission (11), stable disease (2) before transplant.

Table 2 shows in detail the type and doses of drugs used for myeloablative conditioning regimens. Total body irradiation was used in only 4 patients at a dose of 12 Gy followed by etoposide 1800 mg/m^2^ in 1 case and 14.4 Gy in 3 patients followed by cytarabine 24 g/m^2^.

**Table 2:** Type and dose of drugs used for conditioning regimen.

N of drugs	Drug	Peg-filgrastim	Filgrastim	Total
**1**	*Cytarabine 24 mg/m^2^	2	1	3
	*Etoposide 1800 mg/m^2^		1	1
	Thiotepa 900 mg/m^2^	3	3	6
**2**	Busulfan 16 mg/kg, Melphalan 140 mg/m^2^	17	11	28
	Carboplatin 1500 mg/m^2^, Etoposide1500–1800 mg/m^2^	2	5	7
	Carboplatin 800 mg/m^2^, Melphalan 140 mg/m^2^	1		1
	Thiotepa 900 mg/m^2^, Melphalan 140 mg/m^2^	1		1
	Thiotepa 900 mg/m^2^, Etoposide 1500 mg/m2	1		1
**3**	Thiotepa 10 mg/kg, Etoposide 1600 mg/m^2^, Cyclophosphamide 7200 mg/m^2^	3	2	5
	Carboplatin 800–1200 mg/m^2^, Etoposide 800 mg/m^2^, Melphalan 140–180 mg/m^2^		3	3
	Carboplatin 1500 mg/m2, Etoposide 1000 mg/m2, Ifosfamide 12 g/m^2^		1	1
	Busulfan 16 mg/kg, Etoposide 900 mg/m^2^, Cyclophosphamide 120 mg/kg		2	2
**4**	BCNU 300 mg/m^2^, Etoposide 800 mg/m^2^, Cytarabine 1600 mg/m^2^, Melphalan 140 mg/m^2^	2		2

* with total body irradiation, 12–14.4Gray.

Patients of the control group were treated with filgrastim for a median of 9 days, range 6–17. In table 3, the comparison of main transplant variables between the 2 treatment groups is shown, ie. type of conditioning regimen, number of CD34+ cells infused, mucosal and infectious morbidity, PMN and PLT engraftment, use and duration of parenteral nutrition, need and duration of antibiotic therapy, time to discharge and mortality rate. Mean time to engraftment was 10.48 days (standard deviation (SD) 1.57) and 10.44 days (SD 2.44) in filgrastim and pegfilgrastim groups, respectively. The mean of the difference is equal to −0.045 (95% CI: −1.1–1.0). Considering that the upper limit is below 3, the primary endpoint was reached and the non inferiority of pegfilgrastim was established.

**Table 3:** No differences were found in the main parameters of transplant outcome according to treatment groups.

	Filgrastim N = 29, (%)	Peg-filgrastim N = 32, (%)	p
**Type of conditioning regimen With TBI**	2	2	-
**Type of conditioning regimen Without TBI, high-dose chemotherapy**			
**>3 drugs**	19 (70.4)	25 (83.3)	0.2
**<2 drugs**	8 (29.6)	5 (16.7)	
**CD34+ infused**			
**Median**	6.7	6.0	0.4
**Range**	3.0–299.6	3.4–78.9	
**PMN engraftment**			
**Yes**	29 (100.0)	32 (100.0)	-
**Time to PMN engraftment** (days)			
**Mean (SD)**	10.48 (1.57)	10.44 (2.44)	0.3
**Median**	10.0	10.0	
**Range**	8.0–17.0	8.0–23.0	
**PLT engraftment**			
**Yes**	29 (100.0)	32 (100.0)	-
**Time to PLT engraftment** (days)			
**Mean (SD)**	28.10 (17.83)	33.09 (25.51)	0.5
**Median**	22.0	28.5	
**Range**	10.0–84.0	10.0–132.0	
**FUO**			
**No**	6 (20.7)	7 (21.9)	0.9
**Yes**	23 (79.3)	25 (78.1)	
**No. of episodes**			
**Median**	1.0	1.0	0.6
**Range**	1.0–2.0	1.0–2.0	
**Proven infectious**			
**No**	19 (65.5)	23 (71.9)	0.6
**Yes**	10 (34.5)	9 (28.1)	
**TPN**			
**Yes**	29 (100.0)	32 (100.0)	-
**Duration of TPN (days)**			
**Median**	13.0	14.0	0.8
**Range**	5.0–26.0	7.0–30.0	
**Mucositis**			
**No**	5 (17.2)	5 (15.6)	1
**Yes**	24 (82.8)	27 (84.4)	
**Mucositis WHO grade**			
**0–I**	7 (24.1)	13 (40.6)	0.2
**II–IV**	22 (75.9)	19 (59.4)	
**Mucositis duration (days)**			
**Median**	9.5	9.0	0.7
**Range**	3.0–19.0	3.0–23.0	
**Antibiotic therapy**			
**No**	2 (6.9)	3 (9.4)	1
**Yes**	27 (93.1)	29 (90.6)	
**Duration of antibiotic therapy**			
**Median**	14.0	11.0	0.2
**Range**	5.0–41.0	5.0–27.0	
**Time from SCT to discharge**			
**Median**	15.0	15.5	0.7
**Range**	11.0–48.0	12.0–32.0	
**Follow up**			
**Alive**	20 (69.0)	21 (65.6)	0.8
**Died**	9 (31.0)	11 (34.4)	
**Follow-up from SCT (days)**			
**Median**	894	816	1
**95% CI**	261–1323	534–1294	
**Time from SCT to death (days)**			
**Median**	614.0	317.0	0.6
**Range**	71.0–1300.0	157.0–973.0	

TBI, total body irradiation; WHO, World Health organization; TPN, total parenteral nutrition.

Regarding the secondary endpoints, no differences were found in PLT engraftment, episodes of fever of unknown origin (FUO), proven infections, mucositis, days of intravenous antibiotics and parenteral nutrition and days of hospitalization. Both pegfilgrastim and filgrastim were well tolerated and no significant adverse effects were associated with their use. Moreover, no toxic death was reported within the first 100 days post-PBSCT.

**Figure 2:**
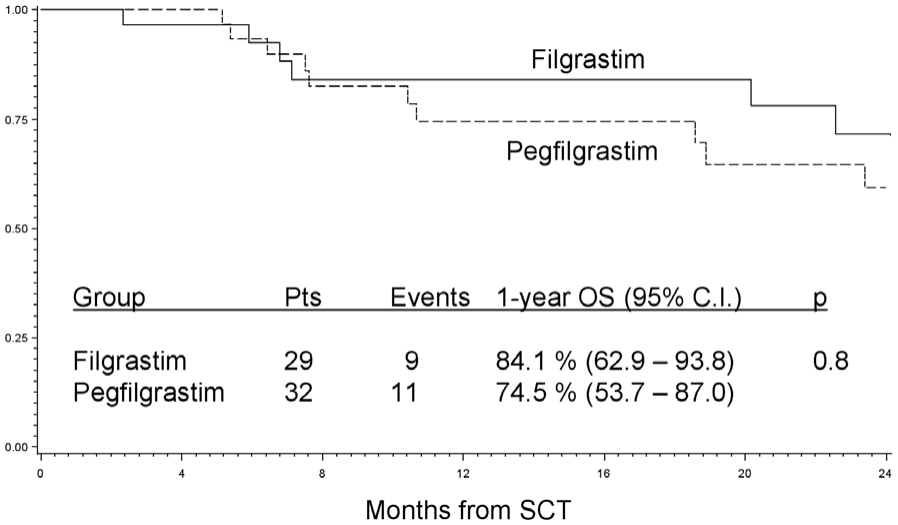
One-year overall survival curve for filgrastim and pegfilgrastim group, respectively. The 1-year overall survival in the in the filgrastim group and in the pegfilgrastim group is shown. No difference was found in the two groups.

After a median follow-up of 2.3 years (95% C.I.: 1.5, 3.3), 41 patients were alive and 20 deaths were observed, 9 in the filgrastim and 11 in the pegfilgrastim group, all due to disease progression. The 1-year overall survival was 84.1% (95% C.I.: 62.9–93.8) in the filgrastim group vs. 74.5% (C.I.: 53.7–87.0) in the pegfilgrastim group, respectively (p = 0.8) (Figure 2).

### 

#### Cost analysis

Considering that no centre had a centralised preparation of supportive drugs and that the discarding of the unused part of the vial was common practice, the cost of treatment was calculated comparing the price of pegfilgrastim and filgrastim vials. On the basis of current acquisition prices in Italy by hospital pharmacies that buy these drugs with a discount >50% off official prices, the costs were 622 euros for a vial of pegfilgrastim (official price 1489,50 euros) and 77.53 euros (official price 127,95 euros) for a vial of the original filgrastim. Given that 1 vial of pegfilgrastim equates to a median of 9 vials of filgrastim found in this study, the median cost of treatment with filgrastim was 698 euros (range 542–1318). This translated into a median saving of 76 euros for every patient treated with pegfilgrastim in addition to the reduced use of health personnel resources for its administration.

## Discussion

The introduction of G-CSF in the late 1980's has radically changed the modality of performing HSCT and of pre-engraftment supportive care. This is true especially for autologous HSCT where the use of G-CSF-mobilized peripheral stem cells and pre-engraftment G-CSF reduced the time of myeloid recovery and, consequently, the incidence of infectious complications and duration of hospital stay [3]. Pegfilgrastim, the pegylated form of filgrastim, is considered equally effective as filgrastim with the advantage of allowing a smooth recovery of neutrophils, and in neutropenic adult patients, reducing the incidence of febrile episodes after chemotherapy [17]. Further advantages are the easier method of administration, one shot of pegfilgrastim compared with daily delivering of filgrastim, and the potential cost-savings because the efficacy of one dose of pegfilgrastim is equivalent to up to 11 doses of filgrastim [18],[19]. The safety and efficacy of pegfilgrastim versus filgrastim after high-dose chemotherapy and autologous HSCT has been assessed in 14 studies of adult patients, 5 of them prospective randomized studies [20]–[24] and the remaining 9 studies being retrospective or prospective with historical-controls [25]–[33]. Pegfilgrastim was demonstrated to be as efficacious as 7 to 12 doses of filgrastim and it achieved faster neutrophil engraftment, with a median gain of one day, and in shortening the duration of febrile episodes. No differences were found for other post-transplant outcomes such as need for transfusion, infection rate, transplant-related mortality, and length of hospital stay [5],[22]. Interestingly, the analysis of costs in 2 randomized trials, one single-centre, double-blind, placebo-controlled, and one multicenter, open-label, showed that the use of pegfilgrastim was less expensive than filgrastim [22],[23]. Pegfilgrastim is still off-label for pediatric patients despite several authors having documented its efficacy and safety for prophylaxis of febrile neutropenia post-chemotherapy and as the mobilizing agent for peripheral blood stem cell collection [6]–[10],[12]–[15],[34]. No data have been published so far on the role of pegfilgrastim as a supportive agent after pediatric autologous HSCT. The main motivation for conducting such a clinical study is the possibility of reducing the costs of supportive post-HSCT drugs [5],[22], considering the increasing demands on health to rationalize and better allocate drug expenditure. For this reason we designed a non-inferiority study between the 2 molecules. Patient groups were comparable for all demographic and clinical characteristics. The homogeneity of the study population is an important issue to avoid the potential bias effect due to type of underlying disease, doses and types of chemotherapy used as conditioning regimen, the main post-HSCT outcomes such as recovery of neutrophils, incidence of febrile episodes, incidence of mucositis, and duration of febrile episodes. A single dose of pegfilgrastim was shown to be not inferior to a median of 9 doses of filgrastim in terms of neutrophil recovery and without any differences for all other variables analysed. Interestingly, as well as this non-inferiority, the use of pegfilgrastim provided a small cost reduction for G-CSF added to reduced health personnel resources in eliminating daily administration of filgrastim. The recent introduction of biosimilars of G-CSF has changed the scenario [35] Biosimilars of G-CSF, that were not available at the time of designing this study, are less expensive than filgrastim and therefore nullify the advantage of pegfilgrastin over filgrastim. No formal study has investigated the cost/benefit ratio of biosimilars over pegfilgrastim as regards unit cost and use of health personnel time.

Another point that is still a matter of debate is the time of initiation of filgrastim, early at day +1 vs. delayed at day +5 or day +7. It is generally accepted that both strategies are equally effective although some studies found an advantage of early G-CSF administration in terms of neutrophil engraftment, number of days of intravenous antibiotics, and length of hospital stay [3]. Despite the fact that this advantage is not completely clear, current guidelines recommend the use of G-CSF from day +1 post-HSCT [36].The delayed initiation of filgrastim is based on the concept that late-committed neutrophilic progenitors responsive to filgrastim are not yet present in the first days after HSCT. As far as pegfilgrastimis concerned, in the literature, the timing of administration ranges from day +1 (most of authors) to day +4 or day +5 [9], [25], [28], [37]. The advantage of a delayed administration of pegfilgrastim is to reduce the clearance by neutrophils obtaining a higher serum level during the period of aplasia. In fact, it is possible that at day +1 the myeloablative effects of conditioning regimen is not complete and the nadir of neutrophils is not achieved yet. To avoid any bias related to a different time of stimulation of myeloid progenitors we decided to start pegfilgrastim and filgrastim at the same time after transplant SCT. Day +3 was chosen because it was considered neither too late to compromise the biologic potential of pegfilgrastim nor too early to compromise the cost/effectiveness of filgrastim. Although this choice could have reduced the potential for a quicker neutrophil recovery with pegfilgrastim, the mean time to neutrophil engraftment was 10 days for both pegfilgrastin and filgrastim which is comparable to that observed in previous studies [3],[5].

In conclusion, this study showed that in pediatric autologous HSCT pegfilgrastim is not inferior to filgrastim for all post-transplant outcomes assessed, with the advantage of lower drug expenditure. The advent of biosimilars nullifies this advantage although prospective randomized studies are needed to compare the costs of 2 different therapeutic choices both in terms of drug expenditure and use of health personnel resources.

## Supporting Information

Protocol S1 (DOC)

Checklist S1 (DOC)
