# Supplementary material for: A Randomized, Non-Inferiority Study Comparing Efficacy and Safety of a Single Dose of Pegfilgrastim versus Daily Filgrastim in Pediatric Patients after Autologous Peripheral Blood Stem Cell Transplant
Source: PLoS One. 2013 Jan 7;8(1):e53252. doi: 10.1371/journal.pone.0053252 (PMC3538773; doi:10.1371/journal.pone.0053252)
Supplement: Protocol S1 — (DOC) [file pone.0053252.s001.doc]

Noninferiority trial 'BETWEEN SINGLE DOSE OF PEG-filgrastim, 100 ug / kg, and filgrastim, 5 ug / kg / day (9 OR MORE' DOSES) IN PEDIATRIC PATIENTS undergoing autologous transplantation of hematopoietic stem cells from peripheral blood

Applicants:

For the SSC-Supportive Treatment, Dr. Simone Cesaro, Oncohematology Padua

In collaboration with the HSC Group and Cellular Therapy group
Participating centers (in alphabetical order of the center)

Prof. Andrea Pession, Oncohematology, St. Orsola Hospital, Bologna
Dr. Fabio Tucci, Oncohematology, Meyer Hospital, Florence
Dr. Cornelio Uderzo, Oncohematology, San Gerardo Hospital, Monza
Prof. Chiara Messina, Oncohematology, Department of Pediatrics, Padua
Dr. Caselli Desireè, Oncohematology, Children's Hospital, Palermo
Professor Franco Locatelli, Pediatric Onco, Policlinico San Matteo, Pavia
Dr. Claudio Favre, Oncohematology, Department of Pediatrics, Pisa
Dr. Franca Fagioli, Oncohematology, Regina Margherita Hospital, Turin
Dr. Marino Andolina, Oncohematology, Institute Burlo Carnation, Trieste
(Extended full address of the centers is available at http://www.aieop.org/stdoc/centri_aieop_20070101.doc)

Statistical analysts
Dr. Roberto Rondelli, Oncohematology, Bologna
Dr. Gloria Tridello, Oncohematology, Padua

Data Collection
Dr. Roberto Rondelli, AIEOP Operation Centre Bologna
 

**Background and rationale**
The high-dose chemotherapy is the mainstay of treatment of many hematologic malignancies and solid in order to exploit the linear relationship between dose and magnitude of response. Unfortunately, the chemotherapeutic agents used in this treatment strategy compromise the ability of cells to divide without distinguishing between healthy normal cells and tumor cells. The bone marrow is a major target of the toxicity of chemotherapy and bone marrow suppression with prolonged severe neutropenia and need for transfusions of red cells and platelets is one of the most frequent events in patients undergoing intensification therapy. For many years it has been demonstrated that the severity and duration of neutropenia after myelosuppressive therapy are related to the risk of systemic bacterial infections requiring hospitalization of the patient and the emergency treatment of the patient with broad-spectrum intravenous antibiotic therapy (Bodey et al 1996; Hughes et al. 1997).
In order to overcome the barrier of the haematological toxicity in the use of doses of chemotherapeutic sovramassimali (myeloablative therapy or megaterapia), already in the 80s has been introduced the infusion of bone marrow stem cells, taken from the patient before the megaterapia, cryopreserved, and subsequently thawed, after treatment with high doses of chemotherapy (autologous transplant).
In the course of the 90 were introduced important changes in the process of autologous transplantation that have allowed to reduce significantly the duration of severe neutropenia and the duration of hospitalization, such as the use of the growth factor granulocyte (G-CSF ) and the use of hematopoietic stem cells harvested from peripheral blood after chemotherapy mobilizing and stimulation with growth factor (Schimitz N et al. 1996). In a study conducted by the Italian Association of Hematology Pediatric Oncology, has been shown that the use of G-CSF allows to accelerate myeloid recovery after transplantation of autologous stem cells from peripheral blood, this being of 11 days, range 10-12 , against a median time of recovery of 14 days, range 12-16, in the control group that had not received the G-CSF (Dallorso, Haematologica 2002).
Recently, it has been introduced a form of pegylated G-CSF (Pegfilgrastim) which, in equal effectiveness, having a greater duration of action simplifies the management of the patient's hematologic. The studies carried out on adults subjected to chemotherapy so far have shown that a single dose of Pegfilgrastim has comparable efficacy to the daily administration of G-CSF (filgrastim) in reducing the duration of severe neutropenia in addition, it significantly reduces the incidence of febrile neutropenia, it simplifies the management of patients with a lower number of daily injections and improve patient compliance (Siena et al, 2003). Also in the adult phase II studies (compared to historical control groups) showed that the Pegfilgrastim is highly effective in the mobilization of peripheral blood stem cells, is equal to filgrastim in reducing the duration of severe neutropenia, but is superior to filgrastim in reducing the incidence of febrile neutropenia and the total length of days of fever in autologous stem cell transplantation. In addition, the drug has shown well tolerated with a safety profile comparable to filgrastim (Staber et al., 2005; Isidori et al. Haematologica 2005; Jagasia et al 2005, Martin et al 2006).
Pegfilgrastim is not currently indicated for the recovery and myeloid cells after transplantation of hematopoietic stem cells.
 
Growth factor granulocyte (G-CSF) and Pegfilgrastim
The G-CSF is the major cytokine that regulates the production of neutrophil granulocytes. The gene for G-CSF is located on chromosome 17q21-22 and coding for a glycoprotein of 19kD and 174 amino acids. The identification of the gene and its characterization has allowed, in the late 80s, to obtain the molecule with the technology of recombinant Escherichia coli (filgrastim) or from cell lines of Chinese hamster ovary (lenograstim). Human use is approved in Europe since 1993 for severe neutropenia in patients receiving chemotherapy or hematopoietic stem cell transplantation, for mobilization of hematopoietic stem cells and for supportive care in patients with AIDS, congenital neutropenia, myelodysplasia, anemia aplastic anemia and leukemia.
Filgrastim, was the first growth factor to be licensed for human use. E 'a protein of 175 amino acids with a group N-terminal methionyl on the end which gives stability to the molecule (Moulinex G, 2004). Filgrastim is a small molecule that is rapidly eliminated by the kidneys. The half-life is approximately 3.5 hours.
The Pegfilgrastim is the pegylated form of filgrastim obtained by covalent binding of a molecule of Polyethylene-glycol with the N-terminal end of filgrastim. PEG is a water soluble polymer, non-toxic, pH-neutral. The addition of PEG to filgrastim determines an increase in the molecular weight and volume of the complex pegfilgrastim (20 kD). These properties determine a modification of the pharmacokinetic properties compared to filgrastim, in particular an increase in the half-life, greater stability in plasma and a slower clearance of the drug. The main change in the pharmacokinetics of pegylated molecule is decreased clearance via the kidneys. The elimination of pegfilgrastim is primarily via the neutrophils, once the molecule is linked to the corresponding receptor, internalized and subsequently degraded. In healthy non-neutropenic, pegfilgrastim when injected subcutaneously in the blood remains high for 9-10 days. In neutropenic patients, the pegfilgrastim reaches a peak after about 24 hours after injection and remains elevated for the duration of neutropenia without daily fluctuation of blood level until, with the recovery of neutrophils, the drug pegylated is removed. This type of clearance "self regulated" allows a more gradual recovery of neutrophils (Waladkhani AR, 2004). Various studies of phase II and phase III trials showed that a single administration of pegfilgrastim of 100 ug / kg is equivalent to 10-11 doses of 5 ug / kg / day of filgrastim in reducing the incidence and duration of severe neutropenia. In addition, a single dose of 6 mg in patients weighing between 46 to 132 kg is effective as a single dose of 5 ug / kg / day of filgrastim (Holmes et al 2002; Green et al 2003; Holmes et al 2002, Waladkhani , 2004). The most frequently reported side effect is mild to moderate bone pain with an incidence similar to that of patients treated with filgrastim.

Objectives of the study
The main hypothesis of this study is that a single dose of pegfilgrastim is not less than 9 consecutive daily doses of filgrastim. This hypothesis stems from the results of an earlier study AIEOP in which the time of engraftment of neutrophils (defined as the first of 3 consecutive days in which the counts of PMNs was greater than 500 x 109 / L) in patients undergoing stimulation with filgrastim after autologous transplantation of HSCs from peripheral blood was between the tenth and twelfth days (Dallorso et al. Haematologica, 2002). Whereas the beginning of the stimulation g. + 3 post-HSCT, it is assumed that a single dose of Pegfilgrastim is not less than 9 doses of filgrastim in obtaining the engraftment of neutrophils

The primary objective of this study is:
1) Time required for myeloid recovery (PMN> 500 x 109 / L x 3 days. Consecutive) after the infusion of peripheral blood stem cells (PBSC);
Secondary objectives are:
2) Number of days. of fever post-PBSC infusion;
3) Number of infection (fever of undetermined origin, infection microbiologically or clinically determined) post-PBSC infusion;
4) Number of days of mucositis after PBSC infusion;
5) Number of days of hospitalization;


Study Design
The criteria for eligibility in the study are:
- Age 0-17
- Autologous transplantation of HSCs collected from peripheral blood after myeloablative conditioning based on one or more of the following drugs: busulfan (14-16 mg / kg), melphalan 140-200 mg/m2, etoposide 40-60 mg / kg, cyclophosphamide 120 -200 mg / kg, thiotepa 10-15 mg / kg, or total body radiation therapy with 12-14.4 Gray
- Minimum available dose of 2 x 10^6^CD34 + / kg to be infused
- Karnofsky or Lansky Play Scale score> 90
- Informed consent of parents (and assent of the adolescent boy)
- Approval of Ethics Committee

During the study will not be allowed the following treatments for potential interaction with growth factor:
- NPT with glutamine
- Therapy Palefermin
- Other experimental treatments

The patients will be randomized to receive either treatment with filgrastim 5 ug / kg / day (maximum dose 300 ug / day), or treatment with pegfilgrastim 100 ug / kg (maximum dose 6 mg single dose), from g. + 3 post-HSCT. Treatment with Filgrastim will be continued until recovery of PMN> 500 x 109/lx 3 days. consecutive. It is instead provided for a second dose of Pegfilgrastim. In case of absence of signs of recovery to the myeloid g. + 14, the patient will be treated at the discretion of the treating physician.

Prophylactic anti-infectious and supportive care for patients in the study
Patients will be hospitalized in single rooms in insulation. It is not required hospitalization in rooms with HEPA filters. There is no prophylactic systemic antifungal and antibacterial. Antiviral prophylaxis with acyclovir is allowed in the HSV + patients before transplantation or diagnosis of disease. The use of non-specific immunoglobulins may be permitted if hypogammaglobulinemia (IgG <4 g / dl) or on the clinical indication.
Transfusion of red cells will be performed for Hb <8 g / dl.
Platelet transfusions from single donor apheresis PLT will be made for values ​​<20.000/mmc.
The use of parenteral nutrition will be made by g. + 1 until recovery is complete myeloid (PMN> 500 for 3 consecutive days), unless otherwise clinically indicated.
 
Costs
Currently pegfilgrastim is not indicated for pediatric patients and for use after transplantation of hematopoietic stem cells, so its use in children should be considered experimental. Considering the difference in purchase price of the 2 study drugs (Pegfilgrastim, 1650.40 euros per vial including VAT, Filgrastim, to 141.77 euros including VAT vial-informant data to pharmaceutical 09/12/06) the dose administered, and the equivalence assumed (1 dose Pegfilgrastim = 9 daily doses of Filgrastim) expenditure per patient would be: Pegfilgrastim 100 ug / kg, 27.5 euro in total; Filgrastim 5 ug / kg / day (2.4 Euros / kg) for 9 doses, 21.6 euro in total. The potential additional expense for each patient would pegfilgrastim of 5.9 per kg of weight (for example, for an average weight of 30 kg, 177 euro; up to a maximum of 354 Euros for a weighing> 60 kg).
In fact, the purchase price by the hospital pharmacies is less than the retail price. In Annex I is shown the cost of a cycle of stimulation with pegfilgrastim and filgrastim, calculated on the purchase price of the 2-drug by the hospital pharmacy of Padua. As can be seen, the cycle of stimulation with pegfilgrastim is slightly lower than with filgrastim. Therefore, it can reasonably be stated that the use of potentially pegfilgrastin does not represent an increase in expenditure pharmaceutical care for the patient.

Statistical Analysis
Sample size
The calculation of the sample size is based on the time required for the recovery myeloid.
The hypothesis of the study is that a single dose of pegfilgrastin is not less than 9 consecutive daily doses of filgrastim. The two regimens will be judged ) is 3 days.Δequivalent if the difference between the two ( It is assumed a standard deviation of the time of engraftment of neutrophils of 3.5 days.
 to 10%,β to 5% and αStaring with a two-sided test is obtained a sample size of 30 subjects per group.

Statistical Methods
The significance level was set at = 0.05) and all tests are two-tailed.α5% (
The variables are described with descriptive statistics such as mean, standard deviation, minimum, maximum and quartiles for quantitative variables and percentages for qualitative ones. The averages and percentages will be accompanied by the confidence interval for the 95%.

Definition of the populations analyzed
"Intention to treat": this population includes all randomized patients who received at least one dose of study treatment and received at least one assessment after randomization.
"For protocol": this population includes all randomized patients who meet all inclusion criteria and no exclusion criteria and completed the study as specified in the protocol.
"Safety": this population includes all randomized patients who received at least one dose of study treatment.

Statistical analysis of the primary efficacy variable
The primary efficacy variable is the time needed for the recovery myeloid and will be analyzed with the Student's t test for independent samples.
Statistical analysis of secondary efficacy variables
The secondary efficacy variables will be analyzed using the Student t test for independent samples.

Randomization
Randomization is expected from the day of admission for HSCT to day + 2.
The randomization of patients will be performed centrally at the AIEOP Operation Centre of Bologna.
The randomization will be done by calling 051.6364667, Monday to Friday (excluding holidays), from 9.00 to 17.00.
Upon request of randomization, the staff of AIEOP CO will be provided the following details:
- Center requires that the randomization
- Name of the patient to randomize
- Date of BMT
At the end of the randomization procedure will be announced on the treatment arm to which the patient was assigned.
Simple randomization will be performed with a ratio of 1:1.
Registration of cases and data analysis
Data from all patients enrolled in the study will be collected by the heads of the centers participating in the study using appropriate data collection sheets that will be sent to them, following discharge Transplant Unit, by post to the AIEOP Operation Centre of Bologna (Dr. ssa Claudia Castellini and Dr. Roberto Rondelli), which will be prepared for an electronic database. This information will then be integrated into the database AIEOP BMT Registry. The evaluation of efficacy and safety will be effected through the use of more appropriate statistical tests.
The personal data acquired through participation in this study will be protected in the manner prescribed by law 675/96 and any other relevant legislation.

  
Bibliography

Bodey GP, Buckley M, SatheYS, Freireich EJ. Quantitative relationships Between circulating leukocytes and infection in Patients with acute leukemia. Ann Inter Med 1966; 64: 328-40

Dallorso S, Rondelli R, Messina C, et al Clinical benefits of granulocyte colony stimulating factor therapy after stem cell transplant hematopietic in children: results of a prospective randomized trial. Haematologica 2002; 87: 1292-8

Green MD, Koebl H, Baselga J, et al. A randomized double-blind, multicenter, phase 3 study of fixed-dose single-administration pegfilgrastim versus daily filgrastim in Patients receiving myelosuppressive chemotherapy. Ann Oncol 2003; 14:29-35

Holmes FA, Jones SE, O'Shaughnessy et al. Comparable efficacy and safety profiles of once-per-cycle pegfilgrastim and daily injection filgrastim in chemotherapy-induced neutropenia: a multicenter dose finding study in women with breast cancer. Ann Oncol 2002; 13: 903-9

Holmes FA, O'Shaughnessy JA, Vukelja S, et al. Blinded, randomized, multicenter study to evaluate abbreviations of administration pegfilgrastim versus daily filgrastim oncer to cycle as an adjunct to chemotherapy in Patients with high-risk Stage II or Stage III / IV breast cancer. J Clin Oncol 2002, 20: 727-31

Hughes WT, Armstrong D, Bodey GP, et al. 1997 guidelines for the use of antimicrobial agents in neutropenic Patients with unexplained fever. Clin Infect Dis 1997; 25:551-73

Isidori A, Tani M, Bonifazi F, et al Phase II study of single pegfilgrastim injection St. adjunct to chemotherapy to furnitures stem cells into peripheral blood of pretreated lymphoma Patients. Haematologica 2005; 90:225-31

Jagasia MH, Greer JP, Morgan DS, et al. Pegfilgrastim after high-dose chemotherapy and autologus peripheral blood stem cell transplant: phase II study. Bone Marrow Transplant 2005; 1-5

Martin M, Pratico G, Messina G, etal Pegfilgrastim Compared with filgrastim after high-dose melphalan and autologous peripheral blood stem cell transplantation in multiple myeloma Patients. Eur J Haematol 2006; (Epub ahead of print)

Molineux G. The design and development of Pegfilgrastim (PEG-rmetHuG-CSF, Neulasta). Curr Pharmaceutical Design 2004, 10:1235-44

Staber PB, Holub R, Linkesch W, et al. Fixed-dose single administration of Pegfilgrastim vs daily Filgrastim in Patients with haematological malignancies Undergoing autologus peripheral blood stem cell transplantation. Bone Marrow Transplant 2005, 35: 889-93

Schimitz N, Linch DC, Dreger P, et al. Randomised trial of filgrastim-mobilized peripheral blood progenitor cell transplantation versus autologous bone marrow transplantion in Patients lymphoma. Lancet 1996; 347: 353-7

Siena S, S Secondino, Giannetta L, et al. Optimising management of neutropenia and anemia in cancer chemotherapy-advances in cytokine therapy. Crit Rew Oncol Hematol 2003; 48S: S39-47

Waladkhani AR. Pegfilgrastim: a recent advance in the prophylaxis of chemotherapy-induced neutropenia. Eur J Cancer Care 2004; 13:371-9
 
"Non-inferiority study 'BETWEEN SINGLE DOSE OF PEG-filgrastim, 100 ug / kg, and filgrastim, 5 ug / kg / day (9 OR MORE' DOSES) IN PEDIATRIC PATIENTS undergoing autologous transplantation of hematopoietic stem cells from peripheral blood"

Principal Investigator: dr. Simone Cesaro, Oncohematology, Padua
Sponsor: Studio spontaneous promoted by the Italian Association of Pediatric Hematology Oncology, Supportive Treatment Discipline Committee and Discipline Committee of Hematopoietic Stem Cell Transplantation and Cellular Therapy

Informed consent of parents (less than patient)

I / We the undersigned / s ........................................................., the patient's parents ............................................................... ........... I am / we were properly informed / from the doctor about the purposes, risks and characteristics of the study in question.
I / We the undersigned / s ............................................................ am / we was / i informed / s from Dr. .................................... ............ about the possibility of access to my data / information or our child.
I / We have / have read and fully understood the form informing the patient / parents. I / We have / have had the opportunity to ask some questions and I / We was / i fully satisfied / the responses obtained. The doctor informed appropriately about the potential risks and possible benefits associated with this study.
I / We have / have had enough time to decide whether to participate in this study. I am / we are aware / s that participation in this study is voluntary and that I / we withdraw my / our participation at any time and without giving an explanation, further withdrawal of consent will not affect my / his future care.
I / We are aware of the insurance coverage provided to me / our child for any effects related to this study.

I / we receive a copy of the form of information to the patient / parents and this informed consent.
I / We agree / we agree to participate in the clinical study mentioned above.


..............................................................................
                                                    place

......................................................................................................
Name of the Doctor asks the consent Date Signature

......................................................................................................
Name of Patient Date Signature

......................................................................................................
Name of Parent Signature Date

......................................................................................................
Name of Parent Signature Date
 

"Non-inferiority study 'BETWEEN SINGLE DOSE OF PEG-filgrastim, 100 ug / kg, and filgrastim, 5 ug / kg / day (9 OR MORE' DOSES) IN PEDIATRIC PATIENTS undergoing autologous transplantation of hematopoietic stem cells from peripheral blood"

Principal Investigator: dr. Simone Cesaro, Oncohematology, Padua
Sponsor: Studio spontaneous promoted by the Italian Association of Pediatric Hematology Oncology, Supportive Treatment Discipline Committee and Discipline Committee of Hematopoietic Stem Cell Transplantation and Cellular Therapy


Module Information for parents and the adolescent patient

Dear Parents,
Dear patient,
This study is a medical research aimed at improving patient care, however, although the result, as in all studies, is not predictable.

What are the premises of this study?
The type of autologous transplant is currently the most used is based on the collection of hematopoietic stem cells circulating in the bloodstream via leukapheresis procedure after the patient undergoes high-dose chemotherapy and stimulation with granulocyte colony stimulating factor. This drug, whose name is filgrastim, has the ability to enhance hematopoietic stem cell division. The hematopoietic cells are then harvested and cryopreserved then infused when the patient is adequately prepared to receive them. In order to reduce the time of hospitalization and the duration of severe neutropenia, filgrastim is also used after the infusion of hematopoietic stem cells to accelerate recovery of bone marrow function and in particular to determine a more rapid increase in the number of neutrophils in the circulating blood.

What is the purpose of this study?
The purpose of this study was to investigate whether a new formulation of pegfilgrastim administered once after 3 days after infusion of autologous hematopoietic stem cells, has the same efficacy of filgrastim administered daily for at least 9 days. Preliminary studies in adults have shown that the use of pegfilgrastim have the same effect, in some cases even better efficacy compared to the results obtained in the past in patients treated with filgrastim, in addition the pegfilgrastim requires a smaller number of injections.
The pegfilgrastim, has a duration of action of many days that persists for the whole duration of the phase of neutropenia and allows a more continuous stimulation of bone marrow stem cells compared to filgrastim.

Study design:
The patients undergoing collection of peripheral blood stem cells after chemotherapy or radiation therapy, high doses (conditioning phase), will be randomized (ie, randomly chosen according to a mathematical model) to receive a single dose of pegfilgrastim of 100 ug / kg or a daily dose of filgrastim is 5 ug / kg subcutaneously (or intravenously) from the third day after infusion of autologous stem cells. The two treatment groups, pegfilgrastim and filgrastim are compared to results obtained in terms of efficacy and tolerability.
There are no specific tests for patients in the study, along with the regular monitoring of clinical hematology. The median follow-up study will include the entire period after the harvest up to 100 days after completion of autologous stem cell transplantation.

What are the known adverse reactions (side effects) of filgrastim and / or pegfilgrastim?
The studies performed to date have shown that the 2 drugs have a comparable safety profile and side effects are usually mild or moderate, affecting not more than 20% of subjects treated and where arise are treatable with common analgesics. The most frequent adverse reactions are bone pain, muscle pain, nausea, injection site pain, increased alkaline phosphatase. Rarer are the skin rash, pruritus, paresthesia, tingling.

Voluntary participation and quality control
Participation in this study is voluntary. Your consent can be withdrawn at any time and without explanation. The withdrawal of consent does not impact on your future medical care.
The study design and conduct of the study are made in accordance with international criteria called "good clinical practice" (GCP).

Therapeutic alternatives
The alternative drug to filgrastim lenograstim which was comparable to filgrastim for tolerability and efficacy. At the moment, are not available in other molecules pegilate of growth factor, in addition to pegfilgrastim.
 
Availability of doctor
You have any questions concerning this study may request clarification from one of the following physicians:

Dr.

Dr.

Dr.


As in all other cases of emergency contact one of the numbers provided to you by your doctor:

Phone __________________

Doctor-Patient Confidentiality / Privacy Policy
All medical personnel involved in his care during this study is obliged to obey established rules on doctor-patient confidentiality and must follow the rules concerning your privacy.
Your data may be used anonymously for scientific publications. If necessary for reasons of monitoring and quality control of data collected, the persons authorized (ie, the Sponsor, responsible for collecting the data center and responsible for collecting data from the study) will have access to meaningful data of the medical records with ' requirement of anonymity with respect to personal data.


Assure that all relevant parts of the study were discussed and that all questions have been answered to the satisfaction of the patient and his parents.


..........................................................................................................
                                                    Location Date

......................................................................................................
Doctor's Name Signature

 
Dear Colleague,

Your assistito______________________ agreed participation in the "" non-inferiority study of 'one single dose of peg-filgrastim, 100 ug / kg, and filgrastim 5 ug / kg / day (9 or more' doses) in pediatric patients undergoing transplantation autologous peripheral blood hematopoietic stem cells "
Principal Investigator: dr. Simone Cesaro, Oncohematology of Padua
Sponsor: Studio spontaneous promoted by the Italian Association of Pediatric Hematology Oncology, Supportive Treatment Discipline Committee and Discipline Committee of Hematopoietic Stem Cell Transplantation and Cellular Therapy
As you know, the type of autologous transplant is currently the most used is based on the use of hematopoietic stem cells collected from peripheral blood by leukapheresis, cryopreserved and then infused after treatment with chemotherapy and / or High-dose radiation (conditioning).
Sincerely
